# The sphingolipid biosynthetic enzyme *Sphingolipid delta8 desaturase* is important for chilling resistance of tomato

**DOI:** 10.1038/srep38742

**Published:** 2016-12-08

**Authors:** Ying Zhou, Lanting Zeng, Xiumin Fu, Xin Mei, Sihua Cheng, Yinyin Liao, Rufang Deng, Xinlan Xu, Yueming Jiang, Xuewu Duan, Susanne Baldermann, Ziyin Yang

**Affiliations:** 1 Key Laboratory of South China Agricultural Plant Molecular Analysis and Genetic Improvement & Guangdong Provincial Key Laboratory of Applied Botany, South China Botanical Garden, Chinese Academy of Sciences, Xingke Road 723, Tianhe District, Guangzhou 510650, China; 2 University of Chinese Academy of Sciences, No. 19A Yuquan Road, Beijing 100049, China; 3 Leibniz-Institute of Vegetable and Ornamental Crops Großbeeren/Erfurt e.V., Theodor-Echternmeyer-Weg 1, 14979 Großbeeren, Germany; 4 Institute of Nutritional Science, University of Potsdam, Arthur-Scheunert-Allee 114-116, 14558 Nuthetal, Germany

## Abstract

The physiological functions of sphingolipids in animals have been intensively studied, while less attention has been paid to their roles in plants. Here, we reveal the involvement of *sphingolipid delta8 desaturase (SlSLD*) in the chilling resistance of tomato (*Solanum lycopersicum* cv. Micro-Tom). We used the virus-induced gene silencing (VIGS) approach to knock-down *SlSLD* expression in tomato leaves, and then evaluated chilling resistance. Changes in leaf cell structure under a chilling treatment were observed by transmission electron microscopy. In control plants, *SlSLD* was highly expressed in the fruit and leaves in response to a chilling treatment. The degree of chilling damage was greater in *SlSLD*-silenced plants than in control plants, indicating that *SlSLD* knock-down significantly reduced the chilling resistance of tomato. Compared with control plants, *SlSLD*-silenced plants showed higher relative electrolytic leakage and malondialdehyde content, and lower superoxide dismutase and peroxidase activities after a chilling treatment. Chilling severely damaged the chloroplasts in *SlSLD*-silenced plants, resulting in the disruption of chloroplast membranes, swelling of thylakoids, and reduced granal stacking. Together, these results show that *SlSLD* is crucial for chilling resistance in tomato.

Sphingolipids are a highly diverse and complex class of lipids that are present in a variety of organisms including animals, plants, fungi, and viruses, and are the major lipids in cell membranes^1^. In plants, sphingolipids make up an estimated 40% of plasma membrane lipids and are enriched in the outer leaflet where they affect membrane integrity and ion permeability^2^.

Sphingolipids are synthesized *de novo* in the endoplasmic reticulum from serine and palmitoyl CoA by serine palmitoyltransferase^3^,^4^. The product of this reaction is reduced to form sphinganine, which contains two hydroxyl groups and has a chain length of 18-carbons with no double bonds. Sphinganine is a kind of long chain base (LCB), which is a unique component of all sphingolipids. The initial LCB can be modified by adding a hydroxyl group at the C-4 position to produce the trihydroxy LCB, 4-hydroxysphinganine. It also can be unsaturated by sphingolipid desaturases. Sphingolipid delta4 desaturase adds a *trans* double bond between the C4 and C5 positions of sphinganine^5^, while sphingolipid delta8 desaturase introduces a *cis* or *trans* double bond between the C-8 and C-9 positions of sphinganine. In mammals, LCB desaturation occurs primarily at the C-4 position, whereas desaturation catalyzed by sphingolipid delta8 desaturase at the C-8 position is predominant in higher plants^6^. For example, approximately 80% of LCBs in *Solanum lycopersicum* (tomato) and *Arabidopsis thaliana* are delta8 unsaturated LCBs^7^.

Besides playing structural roles in cellular membranes, sphingolipids function as bioactive signaling molecules in the regulation of cell growth, differentiation, senescence, and apoptosis in mammals^8^. Compared with the large body of research on the functions of sphingolipids in mammals, there have been relatively few studies on their roles in plants. In plants, sphingolipids have been proposed to play important roles in cell signaling^9^, membrane homeostasis^10^, pathogen resistance^11^, and abiotic stress responses^1^. Sphingolipids and their related enzymes have been shown to play roles in aluminum tolerance^12^, drought acclimation^13^, and resistance to hypoxia^14^. In the model plant *Arabidopsis*, chilling was shown to induce rapid and transient formation of phytosphingosine-phosphate^15^. In another study, *AtSLD* mutants became chlorotic under low temperature, suggesting that there may be a relationship between sphingolipids and the low-temperature response^16^. However, the roles of sphingolipids in chilling resistance are poorly understood. In addition, there are no reports on the relationship between sphingolipids and chilling resistance in economic crops.

Tomato (*S. lycopersicum*) is an important economic crop and a chilling-sensitive plant. It becomes physiologically dysfunctional under low or non-freezing temperatures below about 10 °C to 12 °C^17^. In the present study, the function of tomato sphingolipid delta8 desaturase *SlSLD* in chilling resistance was studied in detail. Suppression of *SlSLD* by virus-induced gene silencing (VIGS) led to a marked decrease in chilling resistance and severe chilling damage to chloroplasts. These findings suggested that *SlSLD* could be a potential target gene in the future to develop chilling-tolerant tomato and other economic crop plants.

## Results

### *SlSLD* was highly expressed in leaves and fruit of tomato subjected to chilling treatment

**Figure 1:**
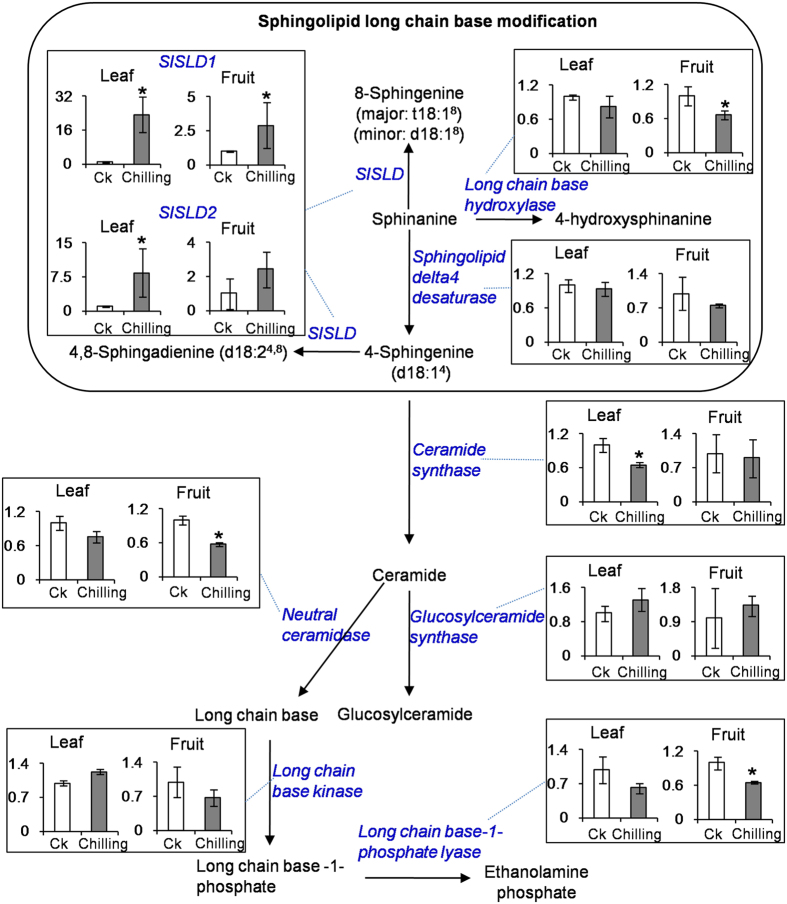
Effect of chilling treatment on the transcript levels of the genes involved in formation and metabolism of sphingolipids in tomato. Data represent the mean value ± standard deviation (n = 3). CK, tomato plants under 25 °C. Chilling, tomato plants under 4 °C for 6 h. The mRNA levels of the genes in the CK were defined as 1. *p ≤ 0.05 compared with CK.

To identify chilling stress-responsive genes related to sphingolipid metabolism, the transcript levels of *SlSLD, long chain base hydroxylase, long chain base-1-phosphate lyase, sphingolipid delta-4 desaturase, neutral ceramidase, long chain base kinase, ceramide synthase,* and *glucosylceramide synthase* were analyzed in tomato exposed to a 6-h chilling treatment. Of these genes, only SlSLD homologs showed increased transcript levels in tomato leaves in response to the 6-h chilling treatment, while the other genes showed non-significant changes or reduced transcript levels (Fig. 1). After the chilling treatment, the transcript levels of *SlSLD1* were increased 23.4-fold in leaves and 2.9-fold in fruit, and the transcript levels of *SlSLD2* were increased 8.4-fold in leaves and 2.4-fold in fruit. These results suggested that SlSLD1 and 2 are important chilling stress-responsive genes involved in sphingolipid metabolism.

### Silencing of *SlSLD* by VIGS affected the chilling resistance of tomato

To further investigate role of *SlSLD* in the chilling resistance of tomato, *SlSLD* was knocked-down by VIGS, and the chilling resistance of the *SlSLD*-silenced plants was evaluated. *Phytoene desaturase*, which encodes an important enzyme in the carotenoid biosynthesis pathway, was used as a positive control for the VIGS system in this study (see Supplementary Fig. S1). The similarity of *SlSLD1* and *SlSLD2* is 66.1% (see Supplementary Fig. S2). Using VIGS based on a *SlSLD2* fragment (see Supplementary Fig. S3), we obtained five silenced plants with knocked-down expressions of both *SlSLD1* and *SlSLD2*. Surprisingly, *SlSLD1* was suppressed more readily than was *SlSLD2* by VIGS (Table 1). We analyzed the major unsaturated LBCs in control and silenced plants. Using UPLC-QTOFMS technique, peak areas of ([M + H]^+^ *m/z* 298.2746) possibly from 4,8-sphingadienine (d18:2) had no significant differences between control and *SlSLD*-silenced plants. However, one peak area of ([M + H]^+^ *m/z* 316.2852) possibly from phytosphingosine (t18:1), which was the major LCB in tomato leaves^7^, reduced in *SlSLD*-silenced tomato plants in contrast to control (see Supplementary Fig. S4). As authentic standards of phytosphingosine (t18:1) are not available on the market, further studies on synthesis of these authentic standards for identifying LCBs in *SlSLD*-silenced tomato plants will provide more evidences concerning involvement of *SlSLD* in metabolism of LCBs.

**Figure 2:**
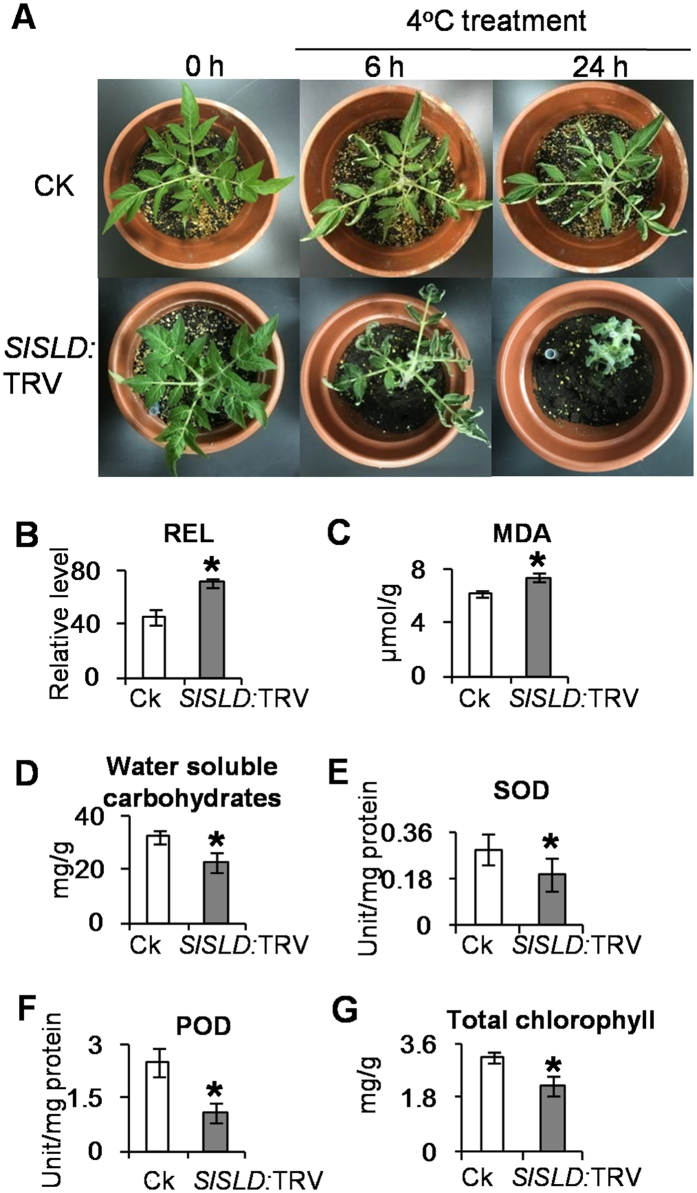
Evaluation of abilities of cold resistance in *SlSLD*-silenced tomato leaves. (**A**) Phenotype of *SlSLD*-silenced tomato after exposure to 4 °C. REL (**B**), MDA content (**C**), water soluble carbohydrate content (**D**), SOD activity (**E**), POD activity (**F**), and chlorophyll content (**G**) were analyzed after chilling treatment for 6 hours. Data represent the mean value ± standard deviation (n = 5). CK, tomato leaves injected with TRV vector. *SlSLD*: TRV, *SlSLD*-silenced tomato leaves. *p ≤ 0.05 compared with CK.

After exposure to 4 °C, *SlSLD*-silenced tomato plants were severely damaged (Fig. 2A), suggesting that *SlSLD* is involved in chilling resistance. Leaves of tomato plants were collected before and after the 6-h chilling treatment, and the following physiological indicators of chilling damage or chilling responses were measured: relative electrolytic leakage (REL), malondialdehyde (MDA), water soluble carbohydrate contents, superoxide dismutase (SOD) activity, peroxidase (POD) activity, and total chlorophyll content. No significant difference was found between control and *SlSLD*-silenced plants before chilling treatment (see Supplementary Fig. S5). However, these physiological indicators of chilling damage suggested higher chilling resistance in control plants after 6-h chilling treatment. Compared with the leaves of control plants, *SlSLD*-silenced tomato leaves showed higher REL, indicating that their cell membranes were more severely damaged by the chilling treatment (Fig. 2B). The MDA content was also higher in *SlSLD*-silenced leaves than in control leaves (Fig. 2C), while SOD activity, POD activity, and water soluble carbohydrate content were lower in *SlSLD*-silenced leaves than in control leaves (Fig. 2D,E,F). These results indicated that silencing *SlSLD* reduced the chilling resistance of tomato. Furthermore, total chlorophyll content was lower in *SlSLD*-silenced plants than in control leaves after the 6-h chilling treatment (Fig. 2G). These results suggested that the chloroplasts may be affected after chilling treatment.

### Exposure to low temperature severely damaged chloroplasts in *SlSLD*-silenced plants

**Figure 3:**
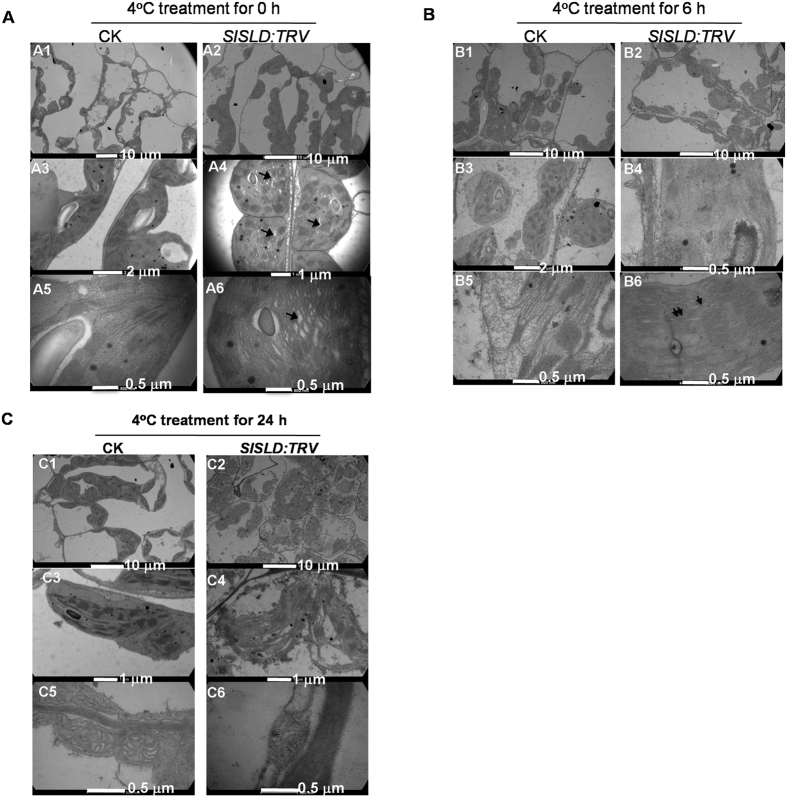
Cell structures of *SlSLD*-silenced tomato leaves during the period of exposure to chilling stress (4 °C). Ultrastructural alteration of the chloroplast after exposure to chilling stress. The chloroplasts morphology in mesophyll cell in CK plant (A1, A3, A5) and *SlSLD*-silenced plant (A2, A4, A6). The normal chloroplasts were observed in CK plant (A1, A3, A5). Although the chloroplasts seem normal under low magnification (A2), swelling of thylakoids was observed (A4, A6 arrows). The chloroplasts morphology in mesophyll cell in CK plant (B1, B3, B5) and *SlSLD*-silenced plant (B2, B4, B6) was observed after chilling treatment for 6 hours. The grana stacks were clearly in CK plant mesophyll cells (B1). The grana stacks were not observed or in regular morphology in *SlSLD*-silenced plant mesophyll cells (B2). The chilling damage of chloroplast was observed in CK plant (B3), however, the grana stacks maintained tight oppression (B5). The grana stacks were disappeared in chloroplast (B4). The grana stacks did not maintain tight oppression (B6). Swelling of stromal thylakoids (arrow) and grana thylakoids (double arrows) were observed. The chloroplasts morphology in mesophyll cell in CK plant (C1, C3, C5) and *SlSLD*-silenced plant (C2, C4, C6) was observed after chilling treatment for 24 hours. The chloroplast was still integrate in CK plant mesophyll cell (C1). The chloroplast was severely destroyed in *SlSLD*-silenced plant mesophyll cell (C2). The detailed morphology of chloroplast in CK plant (C3). The detailed morphology of disrupted chloroplast in *SlSLD*-silenced plant (C4). The mitochondrion was accumulated in CK plant epidermal cell (C5). The accumulation of mitochondrion was not observed in *SlSLD*-silenced plant epidermal cell (C6). CK, tomato leaves injected with TRV vector. *SlSLD:* TRV, *SlSLD*-silenced tomato leaves.

Compared with the CK plant, some chloroplast in *SlSLD*-silenced leaves showed thylakoid swelling (Fig. 3A2 and A4). To determine whether *SlSLD* affected the cell structure of tomato leaves exposed to chilling stress, we investigated the cellular ultrastructure of leaves of plants exposed to 4 °C. After a 6-h chilling treatment at 4 °C, chloroplasts in both *SlSLD*-silenced leaves and control leaves became round (Fig. 3B1 and B2), and the chloroplasts in the *SlSLD*-silenced leaves showed reduced granal stacking (Fig. 3B4) and thylakoid swelling (Fig. 3B6). There was also some cytological damage in control leaves (Fig. 3B3). However, although the chloroplasts in control plants showed an irregular shape, the thylakoids retained a normal appearance (Fig. 3B5). The phenotype of chloroplast was consistent with the chlorophyll degradation in *SlSLD*-silenced plants.

**Figure 4:**
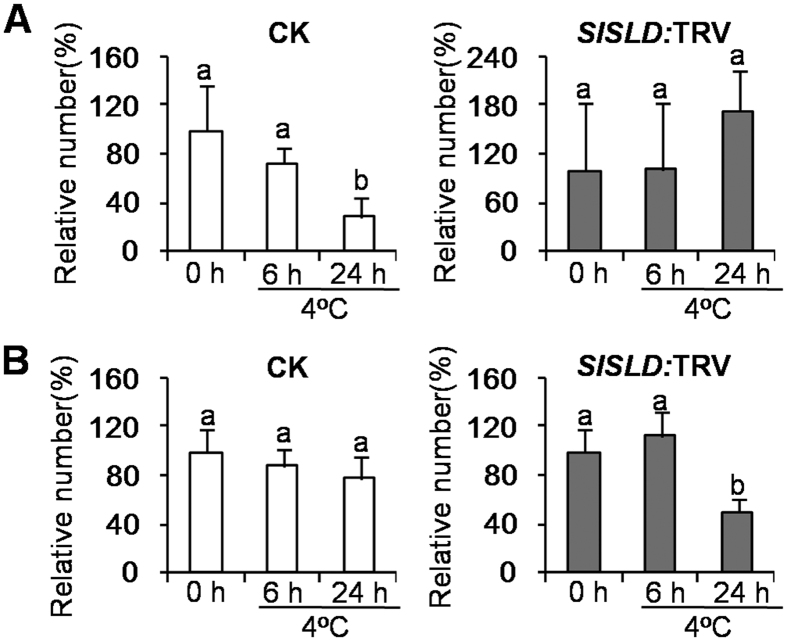
Changes in numbers of starch grain (**A**) and chloroplast (**B**) of *SlSLD*-silenced tomato leaves during the period of exposure to chilling stress (4 °C). The number of starch grain was counted for 10 chloroplasts. The number of chloroplast was counted for 10 cells. Data represent the mean value ± standard deviation. CK, tomato leaves injected with TRV vector. *SlSLD:* TRV, *SlSLD*-silenced tomato leaves. The numbers of either starch grain or chloroplast of untreated plant (4 °C) were defined as 100%.

After a 24-h chilling treatment at 4 °C, the chloroplasts were severely degraded in *SlSLD*-silenced leaves (Fig. 3C2). The number of intact chloroplasts was decreased in *SlSLD*-silenced leaves (Fig. 4B), and those that remained were not surrounded by an extra membrane (Fig. 3C4). In contrast, the chloroplasts in the control plants adapted to chilling stress (Fig. 3C1). The mesophyll chloroplasts regained a normal appearance (Fig. 3C3). In control plants, the chilling treatment resulted in a reduced number of starch grains in chloroplasts (Fig. 4A), and caused mitochondria to accumulate in epidermal cells (Fig. 3C5). This accumulation of mitochondria did not occur in epidermal cells of *SlSLD*-silenced leaves in response to chilling (Fig. 3C6). These phenotypes suggested that the cells consumed stored energy to mount a response to chilling in control plants. Together, all of these results suggested that *SlSLD* is crucial for chloroplast performance upon chilling.

## Discussion

Sphingolipids are structural membrane components of plant cells, and function as potent signaling molecules^18^. Changes in sphingolipid metabolism are linked to a range of environmental stresses^19^,^20^. The results of our study showed that there is a relationship between sphingolipid metabolism and low temperature not only in leaves, but also in the fruit of tomato. Few studies have reported on the responses of enzymes or genes involved in sphingolipid metabolism to low temperature. In this study, *SlSLDs* were the only sphingolipid-related genes showing an increase in RNA abundance in response to low temperature. In a study on the early transcriptional changes in *Beta vulgaris* leaves in response to low temperature, the gene encoding sphingolipid delta8 desaturase was also up-regulated by a cold treatment (−2 °C air temperature for 2 h)^21^. Increased *SlSLD* expression would be beneficial for the production of unsaturated LCBs in tomato. Several studies have shown that the unsaturated sphingolipids content is higher in cold-acclimated or cold-tolerant species^22^,^23^. In another study, an increase in the relative content of unsaturated glucosylceramide (d18:1 c24:0) was positively correlated with increased freezing tolerance in *A. thaliana*^24^. In soybean leaves, the content of *cis* 8-unsaturated sphingolipid long chain base-containing glucosylceramide rapidly increased after a chilling treatment^25^. In the present study, *SlSLD* expression was up-regulated not only in leaves, but also in fruit under a chilling treatment (Fig. 1). These findings suggest that *SlSLD* may also be involved in the response of fruit tissue to low temperature. In this study, therefore, we investigated the function of *SlSLD* in the chilling response of tomato plant using VIGS.

Compared with control plants, *SlSLD*-silenced plants showed higher REL and MDA content under a chilling treatment, indicating that chilling resistance was impaired in the *SlSLD*-silenced plants (Fig. 2B and C). Further analyses showed that the water-soluble carbohydrates content was lower *SlSLD*-silenced plants than in control plants upon chilling (Fig. 2D). Cold acclimation results in the accumulation of soluble carbohydrates and decreased osmotic potential in many plants^26^,^27^. It is possible that the accumulation of low molecular weight cryoprotectants like sucrose help to stabilize the plasmalemma against dehydration-induced changes, and therefore increase freezing tolerance^28^. A correlation between sphingolipids and carbohydrates has been reported in *Fusarium sp*. Compared with untreated *Fusarium sp.*, the treated *Fusarium sp.* contained more sphingolipids and more carbohydrates^29^. However, the relationship between sphingolipids and carbohydrates in plants is still unclear.

Our data demonstrated that sphingolipid metabolism is associated with the reactive oxygen species (ROS)-scavenging system in plants. Experimental evidence from animals and plants has demonstrated that changes in sphingolipid composition affect the ROS-scavenging system. The *alkaline ceramidase* mutant of *Drosophila,* known as *Dacer,* was shown to accumulate C14 and C16-sphingosines, and showed lower ROS production and greater resistance to oxidative stress than the wild type^30^. In tomato, a transgenic line with disrupted sphingolipid metabolism was shown to be sensitive to H_2_O_2_-associated programmed cell death (PCD)^31^,^32^. Our data showed that knocked-down *SlSLD* expression resulted in reduced SOD and POD activities in tomato leaves under chilling stress (Fig. 2E and F). An abrupt increase in H_2_O_2_ levels under cold treatment has been reported in many plants^33^,^34^. There are many reports of a direct correlation between ROS-scavenging ability and cold resistance in plants^35^,^36^,^37^,^38^. The reduced ROS-scavenging ability in *SlSLD*-silenced plants is one of the factors affecting chilling resistance.

Membrane lipid desaturation is a strategy for plants to survive chilling or freezing temperature. Chilling-sensitive plants contain high levels of saturated molecular species of phosphatidylglycerol in their thylakoid membranes, whereas the majority of phosphatidylglycerols are in the unsaturated form in chilling-resistant species^39^. Many studies have reported that changes in lipid unsaturation affect chloroplast morphology. In the *Arabidopsis* fatty acid desaturase mutant *fab1*, the chloroplasts showed an abnormal shape with poorly defined broken envelopes after a 14-day cold treatment at 2 °C^40^. Compared with wild-type plants, the *Arabidopsis* fatty acid desaturase mutants *fadB* and *fadC* contained much fewer thylakoid membranes when grown at 5 °C for 3 weeks^41^. In another study, the absence of enoyl-CoA reductase (ECR) activity affected the very long chain fatty acid (VLFA) composition of sphingolipids^42^. Silencing of *NbECR* resulted in poorly developed thylakoid membranes in *Nicotiana benthamiana*^43^. Together, these results suggested that there is a relationship between sphingolipid composition and thylakoid membrane development and physiology. In *Arabidopsis*, the double mutant of sphingolipid delta8 desaturase, *sld1 sld2,* showed no detectable LCB delta8 unsaturation, with almost completely saturated LCB profiles^16^. It was speculated that silencing *SlSLD* would reduce the unsaturated LCBs content and increase the saturated LCBs content. Cold temperature often results in changes in cell membrane structure. In some cases, the changes in chloroplasts appeared to represent a type of programmed cell death (PCD)^40^,^44^. The saturated LCB d18:0 is considered to be a main contributor to PCD in plant cells^45^. The higher saturated LCBs content in *SlSLD*-silenced plants could result in severe PCD under chilling stress, and thus, could be responsible for the severe chloroplast damage after chilling exposure.

The results of this study showed that two *SlSLD* genes in tomato were activated upon chilling. *SlSLD* is responsible for the biosynthesis of delta8 unsaturated LCBs in plants; thus, higher *SlSLD* gene expression would be beneficial for the biosynthesis of sphingolipids containing delta8 unsaturated LCBs. The gene-silencing experiments in this study demonstrated the essential role of *SlSLDs* in the chilling resistance of tomato, and the importance of *SlSLDs* for the performance of mesophyll cell chloroplasts was demonstrated for the first time. We conclude that *SlSLD* is essential for chilling resistance and normal chloroplast morphology under chilling stress in tomato.

## Methods

### Plant materials and growth conditions

*S. lycopersicum* cv. Micro-Tom plants were grown at 25 °C under a 16-h light/8-h dark photoperiod. For the chilling treatment, 40–50-day-old plants were kept at 4 °C in a chamber with 80% humidity and a 16-h light/8-h dark photoperiod. To produce plants for VIGS, seeds were sown in a 9-mm seedling block and the seedlings were cultivated at 25 °C until the 3^rd^ leaf emerged.

### Analyses of transcript levels of genes involved in sphingolipid formation in tomato leaves and fruits

Total RNA was isolated from leaf or fruit tissue immediately after dissection, using a Quick RNA isolation Kit (Huayueyang, Beijing, China). Reactions were performed using iTaq^TM^ Universal SYBR^®^ Green Supermix (BioRad, Hercules, CA, USA) in a 20-μl reaction mixture containing 10 μl iTaq^TM^ Universal SYBR^®^ Green Supermix (2×), 0.4 μM each specific forward and reverse primer, and 2 μl of a 10-fold dilution of the template. The qRT-PCRs were carried out on a Roche LightCycle 480 (Roche Applied Sciences, Mannheim, Germany) under the following thermal cycling conditions: 95 °C for 30s, 40 cycles of 95 °C for 5s, and 60 °C for 30s. A melting curve analysis was performed at the end of each reaction to verify PCR product specificity. The 2^−ΔΔCt^ method was used to calculate the relative expression level of each gene. The specific gene primers used for target genes and *actin* for qRT-PCR are shown in Table S1. Actin has been used as a reference gene in chilling-treated tomatoes^46^,^47^,^48^,^49^,^50^. Changes in the mRNA level of target gene in each treatment were normalized against that of *actin*.

### VIGS of *SlSLD* in tomato leaves

VIGS was performed as described elsewhere^51^. A 880-bp fragment of *SlSLD2* was amplified by PCR with the forward primer CCGCTCGAGAAAAGTACATTACTGCTGAG and the reverse primer CGCGGATCCAGAAGAGGAAACCAAGTC. The amplified product was cloned into the pTRV2 vector to obtain *SlSLD2*-pTRV2. Then, *SlSLD2*-pTRV2 was transformed into *Agrobacterium* GV3101. After growing *Agrobacterium* cells overnight, the cells containing *SlSLD2*-pTRV2 or pTRV1 were collected. The OD_600_ was adjusted to 0.4 for pTRV1 and to 0.2 for *SlSLD2*-pTRV2. After incubating at room temperature for 6 h, the *Agrobacterium* cultures containing pTRV1 and *SlSLD2*-pTRV2 (1:1) were mixed. The resultant *Agrobacterium* was introduced into leaves of tomato seedlings at the 3^rd^-leaf stage using vacuum infiltration. Plants inoculated with pTRV1 and pTRV2 were used as control plants. The plants were grown at 21 °C, and the gene transcript levels were confirmed at 30 days after VIGS by qRT-PCR.

### Evaluation of chilling resistance of tomato leaves

We measured REL as described elsewhere^52^. Briefly, tomato leaf samples (50 mg) were harvested after the 6-h chilling treatment, and washed in distilled water to remove electrolytes from the leaflet surface. Then, the samples were each placed in a 50-ml centrifuge tube in 10 ml distilled water, and left at room temperature for 22 h. The electrical conductivity of the solution in each tube was measured (R1) using a conductivity meter (Jingke DDS-307A, Shanghai, China) and then the tubes were incubated in boiling water for 30 min. After cooling to room temperature, the electrical conductivity of each tube was re-measured (R2) to determine the maximum electrolyte leakage possible. Then, REL was calculated using the following equation: REL = R1/R2 × 100%.

The MDA content was measured as described elsewhere^53^. Fresh tomato leaf samples (100 mg) were homogenized with 2.5 ml trichloroacetic acid, and then the mixture was centrifuged at 4,000 *g* for 10 min. Then, 200 μl supernatant was mixed with equal volume of 0.6% (v/v) 2-thiobarbituric acid and the mixture was boiled for 30 min. The absorbance of solution was measured at 450 nm, 532 nm, and 600 nm by a spectrophotometer (Epoch Take3, Biotek Instruments, Winooski, VT, USA). The MDA concentration was calculated as follows: MDA concentration (μmol/l) = 6.45 × (OD_532_ − OD_600_) − 0.56 × OD_450_.

The water-soluble carbohydrates content was determined as described elsewhere^54^. Briefly, fresh tomato leaf samples (100 mg) were extracted with 2.5 ml boiling water for 1 h, and then 100 μl of the extract was added to 400 μl 0.2% anthrone in concentrated sulfuric acid. The reaction mixture was incubated in boiling water for 5 min. The absorbance was measured at 630 nm using a spectrophotometer (Epoch Take3). Serial dilutions of glucose were used to generate a standard curve, which was used to calculate the concentration of water soluble carbohydrates in each sample.

To determine SOD activity, fresh leaf samples (0.1 g) were homogenized with 50 mM phosphate buffer (pH 7.0) and then the mixture was centrifuged at 12000 *g* for 20 min. The supernatant served as the protein extract. The SOD activity of the crude soluble protein extract was measured spectrophotometrically with a total SOD activity assay kit WST-8 (Beyotime, Haimen, China) according to the manufacturer’s instructions.

The method to measure POD activity was as described elsewhere^55^. The reaction mixture contained 10 μl soluble protein crude extract as described above, and 190 μl 0.12% H_2_O_2_ (dissolved in 0.56% (w/v) guaiacol). Phosphate buffer was used as the blank. The absorbance at 470 nm was recorded every minute. One unit of enzyme activity was defined as increase in 1 at OD_470_ per min. The specific activity of POD was expressed as unit/mg protein.

The total chlorophyll content was determined as described elsewhere^56^. 0.02 g of leaves was extracted with 2 ml 80% acetone overnight under dark followed by centrifugation at 8000 *g* for 10 min. The total chlorophyll content content was calculated as follows: total chlorophyll content (mg/g) = (17.90 × OD_647_ + 8.08 × OD_665_) × volumn (ml)/1000/weight (g).

### Observation of tomato leaf cell structure

Tomato leaves were cut into approximately 1-mm × 2-mm pieces and fixed in 0.1 M phosphate buffer (pH 7.2) containing 2% (v/v) glutaraldehyde and 2.5% (v/v) paraformaldehyde. After washing six times with 0.1 M phosphate buffer, the leaf samples were fixed in 1% (w/v) osmium tetroxide for 4 h and then washed with 0.1 M phosphate buffer. The fixed leaf samples were dehydrated and embedded in EPON812 resin in flat molds. Ultrathin sections (80 nm) were cut by an ultramicrotome (Leica UC7, Leica Microsysteme GmbH, Wetzlar, Germany), stained with 4% (w/v) uranyl acetate and 2% (w/v) lead citrate, and then observed under a transmission electron microscope (FEI Tecnai^TM^ G2 Spirit Bio Twin, Hillsboro, OR, USA) operating at 100 kV.

### Analyses of LCBs in tomato leaves by UPLC-QTOFMS

Sphingolipids were hydrolyzed using the reported method^7^,^57^. 500 mg (fresh weight) finely powdered samples were hydrolyzed with 2 ml dioxane and 2 ml of 10% (w/v) barium hydroxide octahydrate in water. Samples were hydrolyzed for 16 h at 110 °C. 4 ml of 2% (w/v) ammonium sulfate and 2 ml diethylether were added after hydrolysis. The upper phase was dried under nitrogen and dissolved in 200 μl of methanol. LCBs species were detected by UPLC-QTOFMS (ACQUITY UPLC TMI-Class Xevo G2-XS QTOF, Waters Corp., Milford, MA) using a ACQUITY HSS T3 column (2.1 × 100 mm, 1.8 μm) in positive mode. The mobile phases consisted of water/formic acid (1000/1, v/v) as solvent A and methanol/acetonitrile (3/2, v/v) as solvent B^58^. Elution was started under isocratic conditions of 40% of solvent B for 0.5 min, and followed by increase of solvent B to 70% at 1 min, then increased to 80% at 13 min and subsequently increased to 100% of the solvent B at 14 min and held for 2 min. Flow rate: 0.3 ml/min; Column temperature: 40 °C; Capillary voltage: 2.7 kV; source temperature: 100 °C; desolvation temperature: 350 °C; cone gas flow: 50 l/Hr; desolvation gas flow: 600 l/Hr.

### Statistical analysis

The significance of differences between treatments was calculated by Student’s *t*-test. A probability level of 5% (*p* ≤ 0.05) was considered as significant.

## Additional Information

**How to cite this article**: Zhou, Y. *et al*. The sphingolipid biosynthetic enzyme *Sphingolipid delta8 desaturase* is important for chilling resistance of tomato. *Sci. Rep.* **6**, 38742; doi: 10.1038/srep38742 (2016).

**Publisher's note:** Springer Nature remains neutral with regard to jurisdictional claims in published maps and institutional affiliations.

## Supplementary Material

Supplementary Data

## Figures and Tables

**Table 1:** Expression of *SlSLD* mRNAs in leaves of VIGS-silenced tomato plants.

	Relative expression level*	
	*SlSLD1*	*SlSLD2*
Silenced plant-1	0.0064	0.4666
Silenced plant-2	0.0036	0.4119
Silenced plant-3	0.0048	0.3897
Silenced plant-4	0.0032	0.2449
Silenced plant-5	0.0031	0.1340

^*^The expression levels of genes in control plant was defined as 1.
