# Supplementary material for: The sphingolipid biosynthetic enzyme Sphingolipid delta8 desaturase is important for chilling resistance of tomato
Source: Sci Rep. 2016 Dec 8;6:38742. doi: 10.1038/srep38742 (PMC5143999; doi:10.1038/srep38742)
Supplement: Supplementary Data [file srep38742-s1.pdf]

Title: The sphingolipid biosynthetic enzyme *Sphingolipid delta8 desaturase* is important for chilling resistance of tomato

Authors: Ying Zhou [1,†], Lanting Zeng [1,2,†], Xiumin Fu [1], Xin Mei [1], Sihua Cheng [1,2], Yinyin Liao [1], Rufang Deng [1], Xinlan Xu [1], Yueming Jiang [1,2], Xuewu Duan [1,2], Susanne Baldermann [3,4], Ziyin Yang [1,2 *]

***Affiliation*:**

[1] *Key Laboratory of South China Agricultural Plant Molecular Analysis and Genetic Improvement & Guangdong Provincial Key Laboratory of Applied Botany, South China Botanical Garden, Chinese Academy of Sciences, Xingke Road 723, Tianhe District, Guangzhou 510650, China*

[2] *University of Chinese Academy of Sciences, No.19A Yuquan Road, Beijing 100049, China*

[3] *Leibniz-Institute of Vegetable and Ornamental Crops Großbeeren/Erfurt e.V., Theodor-Echternmeyer-Weg 1, 14979 Großbeeren, Germany*

[4] *Institute of Nutritional Science, University of Potsdam, Arthur-Scheunert-Allee 114-116, 14558 Nuthetal, Germany*

[*] Corresponding author. Ziyin Yang, Tel: +86-20-38072989; Email address: zyyang@scbg.ac.cn.

[†] These authors contributed equally to this work.

Supplementary Information

Table S1 Primers of qRT-PCR used in this study

Gene	ITAG gene ID	Forward primer 5’-3’	Reverse primer 5’-3’
Ceramide synthase	Solyc09g092120	TTCTCTTGGCTCTTATTGGTCTTG	TGTCTCATCACCTTGCTGTTG
Glucosylceramide synthase	Solyc12g008490	GACCGCCATTGTGTAGTGT	GGTGGTGATGTAATAAGCCTCT
Sphingolipid delta 8 desaturase-1	Solyc08g063090	GTAACAACAATAATCTGCATCTCC	GCCTTAATAGAATGACACAATACG
Sphingolipid delta 8 desaturase-2	Solyc05g050090	GTTCTAATCAGCCCCGCCAAAA	ACTACGAATCGGAATATCGCCACC
Neutral ceramidase	Solyc03g006140	CGGCTGCTGATGTCAACA	GCTCGCAACCTGAAGTGAA
Sphingolipid delta-4 desaturase	Solyc10g009030	TCGCATACTTCTTTGGCTCTTT	ACGGTTGTAGGTTGGAGTAGA
Long chain base hydroxylase	Solyc01g010860	ACAAGCATATCATTCTCAGCATCA	CTCCACCGATAGTGTCAAGTAAC
Long chain base-1- phosphate lyase	Solyc05g015860	GAGTTGCCGAAGTCAGGTT	TCAGATGTAGTGGATTGGTATGTG
Long chain base kinase	Solyc02g079030	GTAATACCCGCAGGAACTTCAAA	GGCAAGCATCAGCACACT
Actin	Solyc03g078400	CAGGTGTTATGGTCGGAATGG	ATGCTCAATGGGATATTTCAAGGT

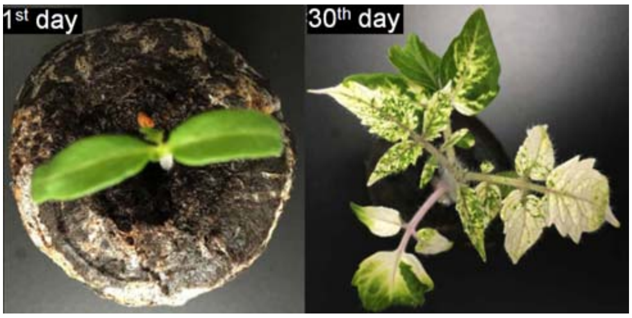

Figure S1 Validation of VIGS using *phytoene desaturase* encoding one of the important enzymes in the carotenoid biosynthesis pathway.

The sense primer used for cloning fragment of phytoene desaturase (PDS) (GenBank accession S36691) was GGATCCGGCACTCAACTTTATAAACC. The antisense primer was CTCGAGCTTCAGTTTTCTGTCAAACC. The cloned PDS fragment was subcloned into pTRV2 vector to get pTRV2-PDS plasmid. The pTRV2-PDS plasmid was transformed into *Agrobacterium tumefaciens* GV3101. The *Agrobacterium tumefaciens* carrying pTRV2-PDS was inoculated into tomato seedling leaves as mentioned in the “Materials and Methods” section.

After the 30 days of VIGS, PDS-silenced tomato leaves showed chlorotic appearance, suggesting that PDS was suppressed successfully.

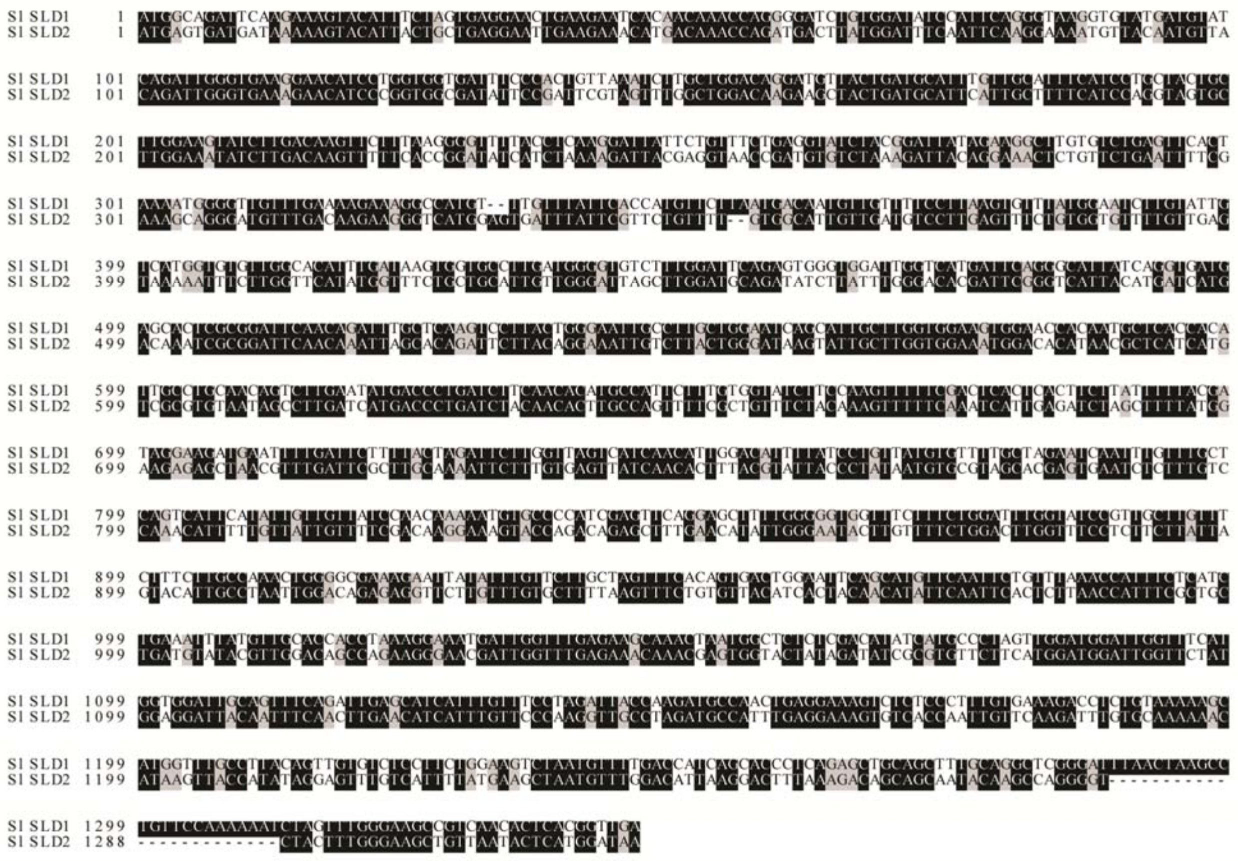

Figure S2 The alignment of SlSLD1 and SlSLD2 sequences.

The sequences were aligned using ClustalX program. The similarity of SlSLD1 and SlSLD2 is 66.1%.

AAAAGTACATTACTGCTGAGGAATTGAAGAAACATGACAAACCAGATGACTTATGGATTTCAATTCAAG
GAAAATGTTACAATGTTACAGATTGGGTGAAAGAACATCCCGGTGGCGATATTCCGATTCGTAGTTTGG
CTGGACAAGAAGCTACTGATGCATTCATTGCTTTTCATCCAGGTAGTGCTTGGAAATATCTTGACAAGTT
TTTCACCGGATATCATCTAAAAGATTACGAGGTAACCGATGTGTCTAAAGATTACAGGAAACTCTGTTCT
GAATTTTCGAAAGCAGGGATGTTTGACAAGAAGGGTCATGGAGTGATTTATTCGTTCTGTTTTGTGGCA
TTGTTGATGTCCTTGAGTTTCTGTGGTGTTTTGTTGAGTAAAAATTTCTTGGTTCATATGGTTTCTGCTGC
ATTGTTGGGATTAGCTTGGATGCAGATATCTTATTTGGGACACGATTCGGGTCATTACATGATCATGACA
AATCGCGGATTCAACAAATTAGCACAGATTCTTACAGGAAATTGTCTTACTGGGATAAGTATTGCTTGGT
GGAAATGGACACATAACGCTCATCATGTCGCGTGTAATAGCCTTGATCATGACCCTGATCTACAACACTT
GCCAGTTTTCGCTGTTTCTACAAAGTTTTTCAAATCATTGAGATCTAGCTTTTATGGAAGAGAGCTAACG
TTTGATTCGCTTGCAAAATTCTTTGTGAGTTATCAACACTTTACGTATTACCCTATAATGTGCGTAGCACG
AGTGAATCTCTTTGTCCAAACATTTTTGTTATTGTTTTCGACAAGGAAAGTACCAGACAGAGCTTTGAAC
ATATTGGGAATACTTGTTTTCTGGACTTGGTTTCCTCTTCT

Figure S3 The sequence used for VIGS.

The sequence used for VIGS was amplified by RCR based on the SlSLD2 gene sequence.

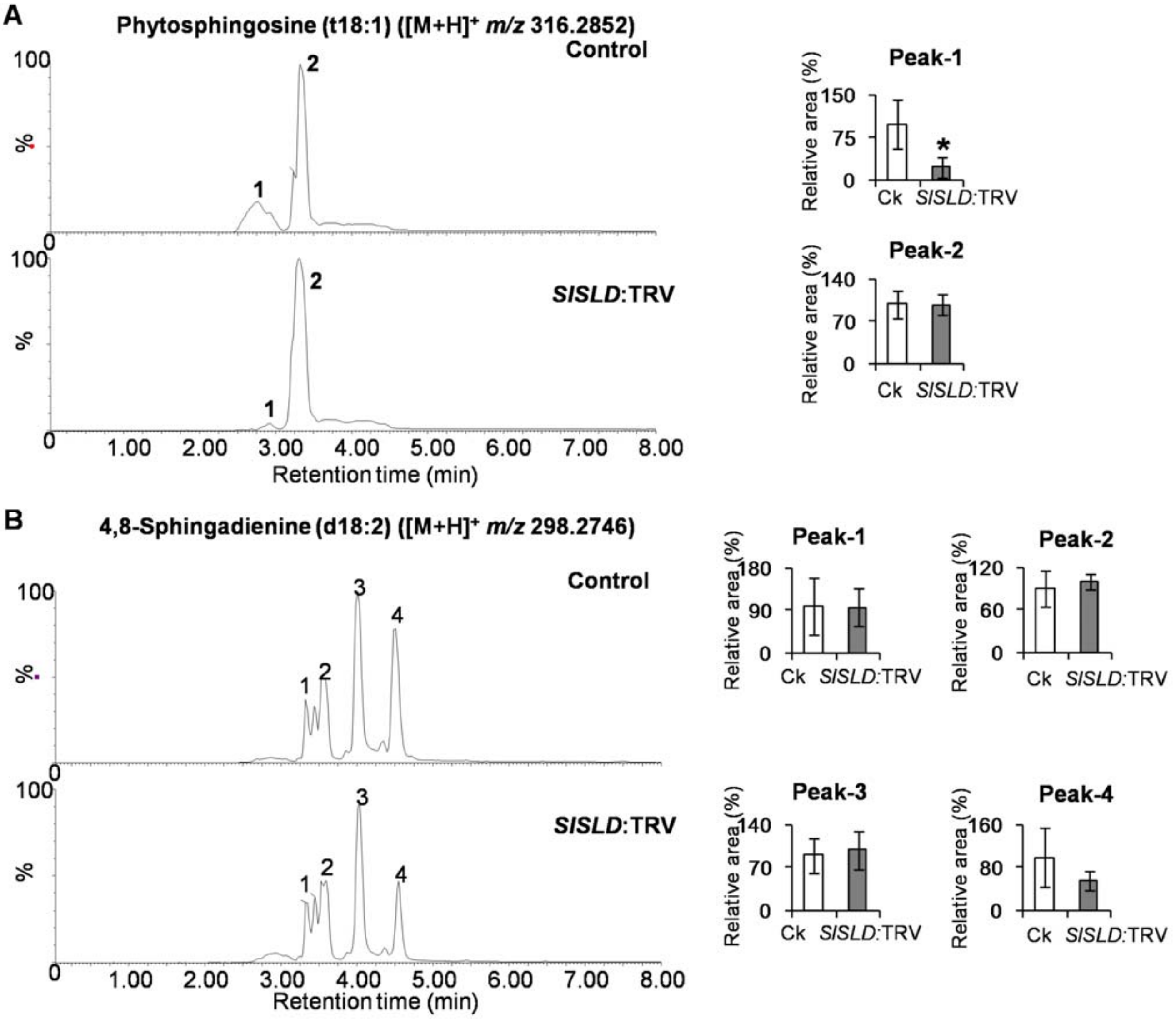

Figure S4 Analyses of the major unsaturated LBCs in control and silenced plants by UPLC-QTOFMS. Control, tomato leaves injected with TRV vector. *SlSLD:* TRV, *SlSLD*-silenced tomato leaves. (A) $[M+H]^+$ *m/z* 316.2852 was possibly from phytosphingosine (t18:1). The peak area of control was defined as 100%. Data represent the mean value ± standard deviation (n=3). *, $p \leq 0.05$. (B) $[M+H]^+$ *m/z* 298.2746 was possibly from 4,8-sphingadienine (d18:2). The peak area of control was defined as 100%. Data represent the mean value ± standard deviation (n=3).

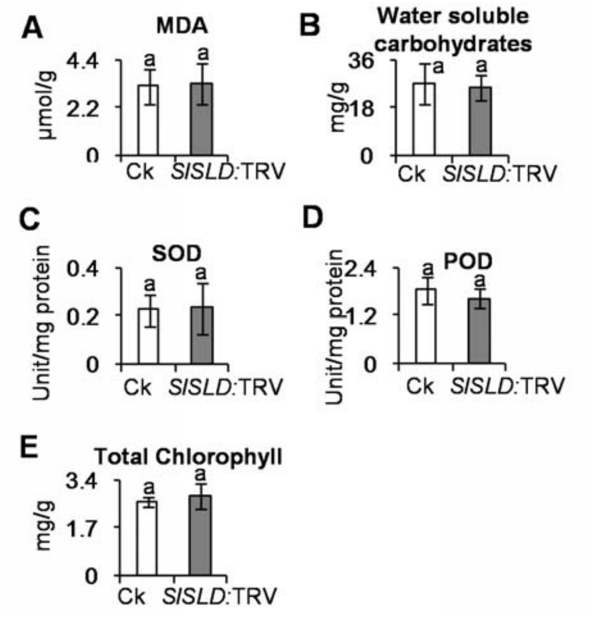

Figure S5 Evaluation of MDA content (A), water soluble carbohydrate content (B), SOD activity (C), POD activity (D), and total chlorophyll content (E) of control and *SlSLD*-silenced tomato leaves before chilling treatment.

Data represent the mean value ± standard deviation (n=4). CK, tomato leaves injected with TRV vector. *SlSLD*: TRV, *SlSLD*-silenced tomato leaves. The same letter indicated no significant difference between Ck and *SlSLD*: TRV.
